# Marked Atrophic Changes of the Brain in a Patient with Subacute Sclerosing Panencephalitis

**DOI:** 10.7759/cureus.1585

**Published:** 2017-08-21

**Authors:** Faizan Yasin, Salman Assad, Muhammad Nadeem, Mehr Zahid

**Affiliations:** 1 Neurology, State University of New York at Buffalo; 2 Department of Medicine, Shifa International Hospital, Islamabad, Pakistan; 3 Surgery, Nishtar Hospital, Multan, Pakistan 60000.; 4 Internal Medicine, University of Lahore, Lahore, Pakistan

**Keywords:** subacute sclerosing panencephalitis, measles virus, seizure

## Abstract

Subacute Sclerosing Panencephalitis (SSPE) is a debilitating disorder associated with the measles infection in childhood. It is a very rare manifestation in children. It usually presents with measles before the age of two. We report a similar case of SSPE in a 14-year-old girl who developed this life-threatening condition in spite of receiving the measles vaccination. Despite the vaccination, the patient had suffered from measles before the age of two. This highlights the dilemma of ineffective vaccinations in developing countries. We also describe the radiologic features of SSPE in this patient, with marked atrophy seen in the occipital region following hyperintensities noticed at a relatively earlier stage.

## Introduction

Subacute sclerosing pan encephalitis (SSPE) is a life-threatening condition that occurs several years after a child suffers from the measles infection [1]. It is believed to occur as a result of a mutation of the virus itself or an excessive immune response to the virus, which results in brain inflammation, followed by necrosis. This condition is associated with a worse prognosis and, usually, death occurs in three years' time [2]. In the earlier stages of the disease, psychiatric manifestations, such as behavioral changes, mood swings, depression, and cognitive decline, are noted. Later on, the patient begins to experience myoclonic jerks, twisting motions, and muscle spasms [3]. A difficulty in regulating blood pressure and temperature usually ensues, and death follows. There is no effective cure for this condition; however, if it is diagnosed at an earlier stage, it can sometimes be medically treated. Magnetic resonance imaging (MRI) changes are not definite and vary from person to person. They can range from hyperintensities in the parietal and occipital region, brain stem lesions, and marked atrophy, causing prominence of sulci. Diagnosis is usually done on the basis of MRI findings, electroencephalogram (EEG) findings, and the detection of anti-measles IgG antibodies in the cerebrospinal fluid (CSF) or serum [4].

## Case presentation

**Figure 1:**
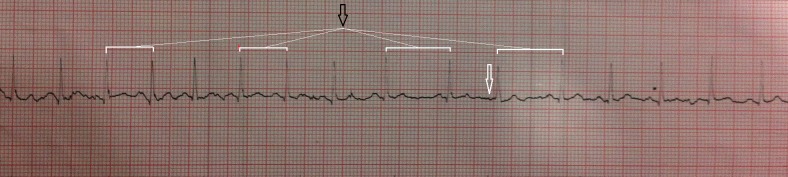
ECG findings Electrocardiogram (ECG) showing atrial fibrillation - irregularly irregular R-R intervals. Loss of P waves also seen (white arrow).

A 14-year-old female presented to the emergency department at Ittefaq Hospital (Trust) in Lahore, Pakistan, with a complaint of an altered level of consciousness for two days and a history of fits for six months, the most recent episode occurring two days ago. Vitals were monitored and revealed a very low blood pressure of 90/50 mm Hg, a heart rate of 116/min, a temperature of 98 ⁰F, and an oxygen saturation of 98%. The blood glucose level was 135 mg/dL. An electrocardiogram (ECG) and arterial blood gases (ABGs) were ordered and the patient was admitted to the intensive care unit (ICU) under the care of a neurologist. The ECG revealed atrial fibrillation (Figure 1).

On admission to the ICU, the patient received IV diazepam 2 mg, which was repeated five hours later; a 5 mg dose was given again after three hours. The Glasgow Coma Scale (GCS) was 10/15 and vital signs were normal. She was started on intravenous (IV) ceftriaxone 2 g two times daily, IV vancomycin 1 g two times daily, IV acyclovir 750 mg three times daily, IV dexamethasone three times daily, IV sodium valproate 500 mg two times daily, IV omeprazole 40 mg once daily, IV diazepam 5 mg in case of fits, and IV phenytoin 300 mg at night.

**Figure 2:**
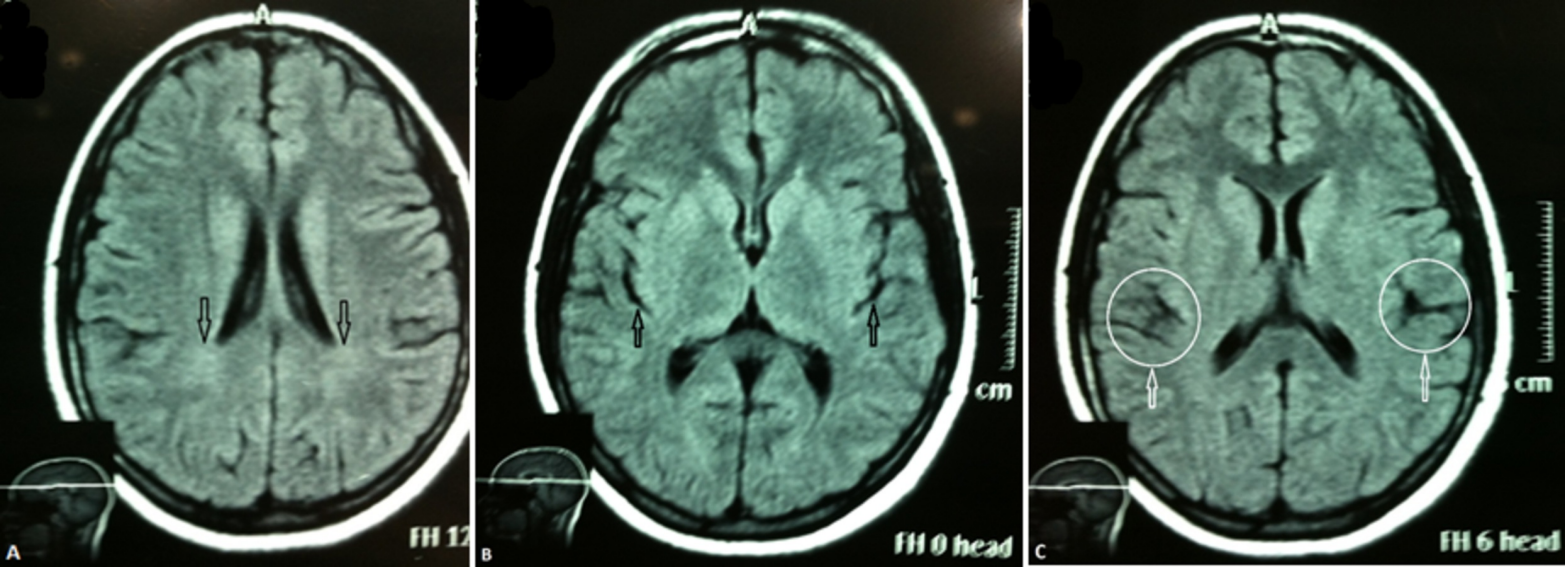
MRI brain findings Magnetic resonance imaging (MRI) showing moderate hyperintensity in the parietooccipital region bilaterally (arrows in A). Cortical atrophy is present, signified by markedly enlarged sulci (arrows in B). Enlarged sulci representing cortical atrophy signified by encircled areas in C.

The patient presented with an altered mental status, generalized muscle rigidity, and episodes of urinary and fecal incontinence that started two days earlier. Previously, the patient was in a healthy state till six months prior, when she suddenly developed a seizure while at her school. This episode was witnessed by some of her class fellows who revealed that it involved twisting of her hands and raised eyelids. This episode lasted approximately 40-50 seconds and was followed by the patient losing consciousness. There was no frothing or tongue biting during that seizure episode. Thereafter, the patient started to experience behavioral changes that were noticeable by her family members. She would experience bouts of anger, frequent mood swings, and depression. There were instances where she would just drop things like a cup of glass. Cognitive impairment also ensued, as she had started putting her hands into food that was being cooked, indicating impaired judgment, and was unable to remember recent happenings, indicating short-term memory loss. The symptoms were gradually progressive, as she witnessed another episode of fits, this time involving both her hands and feet. She was taken to a private hospital facility where she was referred to a neurosurgeon who prescribed her valproate sodium and referred her to a psychiatrist. Over the last three months, her speech had slowed and blurred, eyes had protruded, and she developed ataxia. She also experienced difficulty in swallowing and had repeated episodes of myoclonic spasms/jerks. An MRI brain (Figure 2A-2C) was advised by the neurosurgeon two months ago, which revealed moderate hyperintensity in the parietooccipital region bilaterally (arrows in A) and cortical atrophy, signified by markedly enlarged sulci (arrows in B and C). This was consistent with a relatively earlier stage of SSPE.

Her condition worsened as she continued to experience myoclonic spasms/jerks along with generalized spasticity. She was admitted to another hospital facility where a lumbar puncture was done. A CSF analysis showed moderately elevated glucose, mildly elevated protein, eight white blood cell (WBC)/uL with 70% neutrophils and 30% lymphocytes. Antimeasles IgG antibodies in the CSF were elevated. No atypical or malignant cells were seen on cytology. No microorganisms were seen on gram stain, neither was acid fast bacilli seen on ZN stain. The appearance of the CSF was clear and watery. The CBC revealed slightly decreased hemoglobin and increased neutrophils on the differential count. Liver function tests, serum electrolytes, blood urea nitrogen (BUN), and serum creatinine were all normal.

After being discharged from that hospital, the patient now presented at our hospital after her recent episodes of fits. Her mother admitted that she was fully vaccinated after birth. The patient continued to have altered sensorium and would not respond to any commands, suggesting impaired comprehension. On examination, there was no pallor or cyanosis, the respiratory effort was normal, there was no raised jugular venous pressure (JVP), and she had a soft non-tender abdomen, S1 + S2 + 0 heart sounds, and bilaterally down-going toes in the plantar reflex. However, increased rigidity and tone of the whole body was observed. Complete blood count (CBC), liver functions tests (LFTs), urinalysis, serum electrolytes, ABGs, prothrombin time (PT), international normalized ratio (INR), activated partial thromboplastin time (APTT), C-reactive protein (CRP), and erythrocyte sedimentation rate (ESR) were advised. CBC showed slightly decreased hemoglobin, hematocrit, mean corpuscular volume (MCV) and raised red blood cell distribution width (RDW). CRP was raised, serum potassium level was decreased, BUN and serum creatinine were normal, calcium level was slightly decreased, magnesium normal and serum sodium was normal as well. LFTs were normal whereas serum albumin was slightly decreased. PT, APTT, and INR were normal. Urinalysis showed traces of protein and minimal blood. Microscopic urine examination showed 2-3 triple phosphate crystals. The patient denied having a headache or vomiting, however, she appeared confused and had a low-grade fever. She would not communicate with anyone but would sometimes talk to herself and her speech would usually make no sense. 

**Figure 3:**
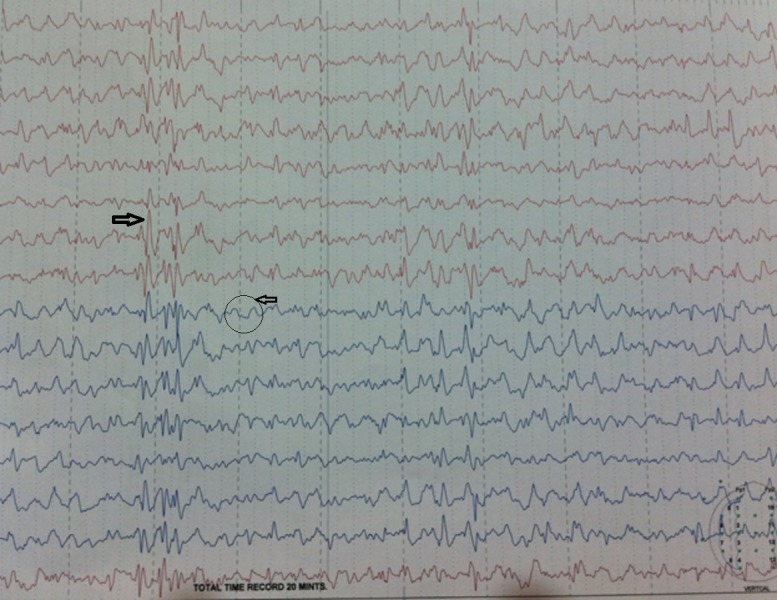
EEG findings Electroencephalogram (EEG) showing a generalized spike and wave pattern or generalized sharp waves (black arrows).

The patient was shifted from medical ICU to the medical ward on her second day of hospital stay. Thereafter, she experienced another episode of tonic clonic seizure that lasted approximately 40-50 seconds and was accompanied by mouth frothing. She was afebrile and vitals were stable. Later that evening she experienced another fit which lasted 30 seconds and was accompanied by a fever of 100 F. Slow IV Phenytoin 1000 mg infusion was started over 30-40 minutes STAT, which was repeated at a dose of 500 mg STAT with slow infusion over 20 minutes. IV diazepam 2 cc was given STAT and IV sodium valproate 1000 mg was given slowly STAT. She was shifted back to the medical ICU. Next day, the patient was prescribed metronidazole 400 mg three times daily (orally), N/G-tube feeding six hourly, IV haloperidol 5 mg for restlessness, levetiracetam 250 mg three times daily (orally), and IV normal saline 500 ml plus IV potassium chloride 40 milliequivalents over four hours STAT. The patient continued to be in a restless state. She was difficult to control, hence, she was confined to her bed by tying her hands and feet to the bed. The patient appeared to be confused with wandering eyes and gaze preference at times towards left (nystagmus) along with jerky movements of the right arm. An EEG was advised. A 32-channel EEG was performed during conscious state. Most of the record revealed a generalized spike and wave pattern or generalized sharp waves (Figure 3).

**Figure 4:**
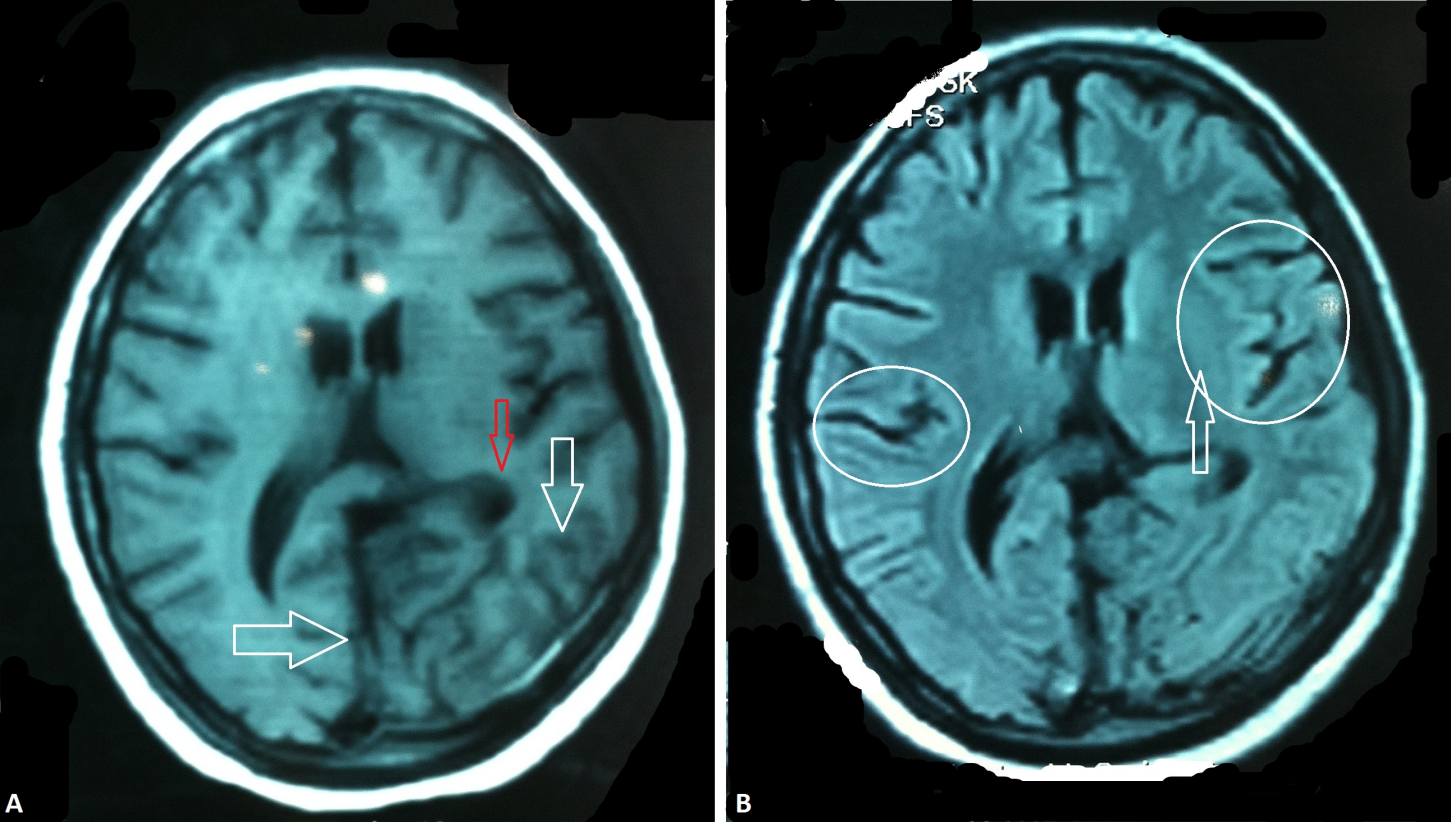
MRI brain without contrast Magnetic resonance imaging (MRI) showing marked atrophy in the parietooccipital region, predominantly in the left occipital region (white arrows in 4A). Sulci appeared to have widened (white circles in 4B). The posterior horn of the left lateral ventricle appeared distorted and compressed (red arrow in 4A).

The patient again had a seizure episode over the next couple of days’ time. An MRI brain without contrast and a lumbar puncture were advised but were refused by the patient’s attendants. Left-hand twitching was also noticed. Betamethasone 25 mg two times daily (orally) was added to her regimen along with Lamotrigine 25 mg (orally) and IV Lacosamide 100 mg. The patient continued to have seizures, this time involving both lower limbs. She was afebrile and vitally stable. The patient was started on IV valproate sodium 500 mg three times daily, IV levetiracetam 500 mg three times daily, IV lacosamide 100 mg once daily, oral omeprazole 20 mg one capsule daily, IV phenytoin sodium 250 mg once daily, topiramate 50 mg two times daily (orally), donepezil 5 mg once daily (orally), methylcobalamin three times daily (orally), multivitamins plus calcium (orally), IV ceftriaxone 2 g two times daily, alfacalcidol, acetyl L-carnitine once daily (orally), and pantoprazole once daily (orally). An MRI brain without contrast (Figure 4A-4B) was done, which revealed marked atrophy in the parietooccipital region, predominantly in the left occipital region (white arrows in 4A). Sulci appeared to have widened (white circles in 4B). The posterior horn of the left lateral ventricle appeared distorted and compressed (red arrow in 4A).

The lamotrigine dose was changed to 7.5 mg and physiotherapy was ordered. The patient’s attendants were informed of the fatal outcome of this condition, which was quite hard for them to absorb. Over the next day, the patient was fully conscious and responsive to commands, however, she would continue to experience fits again and was finally discharged on oral anticonvulsants.

## Discussion

SSPE is a life-threatening condition affecting children who suffer from measles in early childhood. MRI scans have proven to be a method of sensitive investigation in detecting abnormalities in SSPE, however, such abnormalities are not well defined in the literature [5]. Most commonly, hyperintensities on T2-weighted images are seen in patients having SSPE. Our patient had a similar presentation on her earlier MRI, which later progressed to marked atrophy in the occipital region. It has been decades since the cold box was designed for the transport and storage of vaccines; however, the effectiveness of such measures has been somewhat unsuccessful in developing countries. This is largely due to the fact that there is a lack of electric power and many of these countries have a hot, tropical climate. These are some reasons that have led to much of the cold chain equipment being unsuitable for use in developing countries [6]. This might well be the case in our patient, where the mother of the patient claimed that her child was fully vaccinated on the due dates but she still suffered measles before she turned two years' of age. This might well be attributed to the lack of effectiveness of the measles, mumps, rubella (MMR) vaccination, which continues to be quite a dilemma in our part of the world. In addition, neurologists and psychiatrists must be aware of this disease, especially of the fact that initial presentation suggests that it is most likely a psychiatric problem. Hence, taking a detailed history, including the past medical and vaccination history, is pivotal in ruling it out.

## Conclusions

SSPE is quite a challenging diagnosis for physicians worldwide, largely due to the fact that no well-defined findings on imaging modality are known to be definitive for the condition. Hence, MRI findings, correlated with clinical and EEG findings, are needed to confirm the diagnosis. The incidence of SSPE is, fortunately, decreasing due to effective vaccination; however, sporadic cases still continue to surface, especially in developing countries. Unfortunately, there is no effective cure for the condition, especially if it progresses to the later stages of the disease.
